# Indocyanine green fluorescence-guided laparoscopic central pancreatectomy for complete pancreatic transection trauma: a rare case and literature review

**DOI:** 10.3389/fsurg.2024.1448064

**Published:** 2025-01-13

**Authors:** Xitao Wang, Xiong Teng, Yi Liu, Wei Cheng

**Affiliations:** Department of Hepato-Pancreato-Biliary Surgery, Hunan Provincial People's Hospital (The First Affiliated Hospital of Hunan Normal University), Changsha, Hunan, China

**Keywords:** incodyanine green fluorescence navigation, pancreatic trauma, laparoscopic central pancreatectomy, emergency surgery, parenchyma-sparing resection

## Abstract

**Background:**

Pancreatic trauma is a rare solid organ injury. Conservative treatment is often indicated in patients with no pancreatic duct injury, while patients with high-grade pancreatic damage most often require surgical intervention. Laparoscopic central pancreatectomy (LCP) is a parenchyma-sparing approach and can prevent endocrine and exocrine insufficiency after pancreatic resection. Indocyanine green (ICG) fluoroscopy can help the surgeon assess the blood supply of the target organ.

**Case presentation:**

The case we describe here is a 33-year-old male patient who was transferred to our hospital due to blunt abdominal trauma caused by a car accident. The patient was hemodynamically stable on admission and was diagnosed with isolated pancreatic trauma by a multidisciplinary team that included radiologists, emergency physicians, and pancreatic surgeons. The patient then underwent emergency laparoscopic central pancreatectomy, during which we used ICG fluoroscopy to assess the blood perfusion of the damaged pancreas to determine the extent of resection. The patient developed a biochemical fistula (grade A pancreatic fistula) after surgery, and no other intervention was performed except for continuous drainage. The patient was discharged on postoperative day 13. At the 3-month follow-up, the patient did not present any clinical manifestations of pancreatic endocrine and exocrine insufficiency.

**Conclusion:**

To the best of our knowledge, there have been no reports of ICG-guided emergency LCP for blunt abdominal trauma. In selected patients, emergency LCP is feasible and should be supported by a multidisciplinary team and performed by an experienced pancreatic surgeon with advanced laparoscopic skills.

## 1 Introduction

Pancreatic trauma, the incidence of which is reported to be 0.2%–0.3% in all trauma patients, is infrequent compared to other solid organ injuries of the abdomen (1). However, the lesion can be associated with acute hemorrhage, fistula, pseudocysts, intraabdominal abscesses, and pancreatitis, which contribute to major morbidity and mortality (2). Conservative management is mainly advocated for pancreatic trauma without ductal injuries, whereas more extensive injuries generally require therapeutic operative interventions, even pancreatic resection (3).

For benign lesions of the mid-pancreas, central pancreatectomy (CP), which preserves more normal pancreatic parenchyma to avoid long-term endocrine and exocrine pancreatic insufficiency, is an alternative to standard pancreatic resections such as distal pancreatectomy (DP) (4). In recent years, surgeons have sought to minimize the aggressiveness of pancreatic resection by applying a minimally invasive approach. Potential benefits of reduced postoperative pain, faster recovery, and decreased wound complications have been revealed in laparoscopic central pancreatectomy (LCP) compared with open central pancreatectomy (OCP) (5). However, there is a paucity of literature on LCP in emergency settings.

Indocyanine green (ICG), a water-soluble tricarbocyanine dye approved for clinical use in the 1950s, can be injected into the human bloodstream and becomes fluorescent once excited with specific near-infrared light (NIR, approximately 820 nm). Then, fluorescence can serve as a useful real-time imaging signal to provide detailed anatomical and organ perfusion information during surgery (6).

In this study, we present a rare case of a 33-year-old male patient who received ICG-enhanced fluorescence-guided LCP for complete traumatic transection of the pancreatic neck and body.

## 2 Case presentation

**Figure 1:**
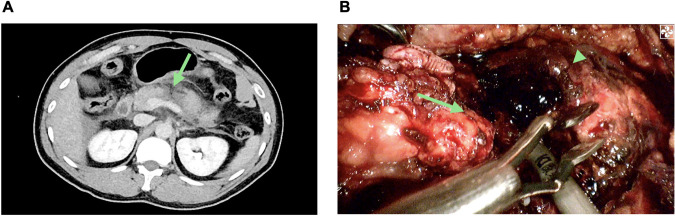
Isolated pancreatic trauma. **(A)** Abdominal computed tomography (CT) showing discontinuity at the junction site of the pancreas neck and body (the green arrow). **(B)** Pancreatic disconnection seen on laparoscopic exploration; proximal pancreatic remnant (the green arrow); distal pancreatic remnant (the green arrow head).

A 33-year-old male patient who had a vehicle crash with a steering wheel impact was brought to the emergency department of a local hospital. The initial computed tomography (CT) showed suspected pancreatic injuries, and after some conservative treatments, including blood transfusion, he was transferred to our hospital. On admission, the patient complained of upper abdominal pain for approximately 10 h. His pulse was 106 beats per minute, and his blood pressure was 150/80 mmHg. On physical examination, his abdomen was diffusely tender with guarding. Laboratory studies revealed normal hemoglobin (141 g/L, reference range: 131–172 g/L) and white cell counts (7.51 × 109/L, reference range: 3.97–9.15 × 109/L) but elevated serum amylase (244.8 IU/L, reference range: 10–160 IU/L) and lipase (513.9 IU/L, reference range: 0–190 IU/L). Abdominal CT suggested pancreatic neck and body parenchymal discontinuity, peripancreatic effusion, and hemoperitoneum (Figure 1A). After a multidisciplinary discussion including radiologists, emergency physicians, and pancreatic surgeons, the patient was initially diagnosed with isolated pancreatic trauma, and therapeutic laparoscopy was suggested.

**Figure 2:**
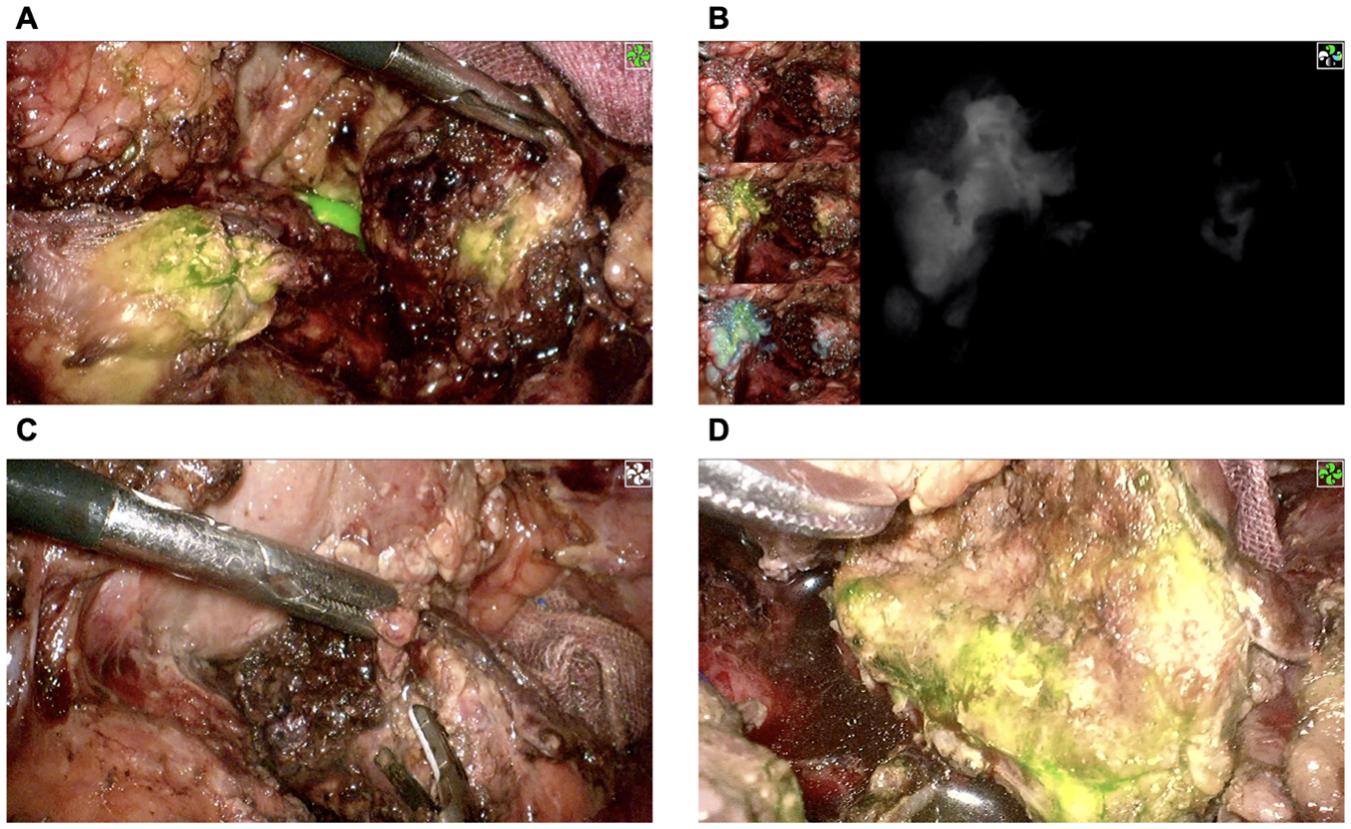
Confirmation of pancreatic stump blood perfusion by ICG fluorescence signaling. **(A**,**B)** The proximal pancreatic stump had significant ICG fluorescence signaling, while the distal pancreatic stump had almost no ICG fluorescence signaling. **(C)** Removal of devitalized distal pancreatic parenchyma. **(D)** Reconfirmation of distal pancreatic stump blood perfusion using ICG fluorescence signaling. ICG, indocyanine green.

The intraoperative investigation found a large amount of abdominal hemorrhage (approximately 1,000 ml), scattered fatty saponification spots on the greater omentum, and complete transection at the junction of the pancreas neck and body (Figure 1B). There was no evidence of other adjacent organ injuries, and intraoperative hemodynamics were stable. Therefore, a laparoscopic central pancreatectomy was planned. To precisely remove the devitalized pancreatic tissue, we used ICG fluorescence imaging to assess blood perfusion in the injured pancreas and thus to determine the extent of central pancreatic resection. ICG (25 mg, Dandong Yichuang Pharmaceutical Co., LTD, China) was diluted with 10 ml normal saline, and then 2 ml was administered through a peripheral vein. The ICG fluorescent signal was identified to confirm the blood flow of the proximal and distal pancreatic stumps (Figures 2A,B). Guided by this, we removed approximately 2 cm of damaged pancreatic parenchyma without fluorescent signals around the transection site (Figures 2C,D). The proximal remnant was oversewn with interrupted nonabsorbable sutures, and a Roux-en-Y pancreaticojejunostomy (PJ) was performed on the distal remnant using modified Blumgart anastomosis. A 6-Fr polyvinyl catheter was inserted into the main pancreatic duct of distal pancreatic stump for external drainage, and four drainage tubes were left next to the proximal stump and the distal pancreaticojejunostomy. The operation time was 386 min, and the estimated blood loss was 100 ml. No blood transfusion was needed during this procedure.

During the hospital stay, a biochemical leak (postoperative pancreatic fistula grade A) was observed, and no further intervention was carried out other than continuous drainage. The patient was discharged from the hospital on postoperative day (POD) 13 and was followed up for 3 months. There was no exocrine or endocrine insufficiency in the patient.

## 3 Discussion

Traumatic injuries to the pancreas account for less than 2% of blunt abdominal traumas, while injuries to the liver, spleen, and kidneys are far more common (7, 8). Approximately 60% of pancreatic injuries are caused by vehicle accidents as a result of impacts with the steering wheel in adults or bicycle handlebars in children (9). Despite its low incidence, pancreatic trauma is a severe solid organ injury, with morbidity rates of up to 60% and mortality rates of more than 20% (10–12). Several factors were clarified to be associated with the poor prognosis of pancreatic trauma (13–16): (1) the deep retroperitoneal location of the pancreas leading to delayed diagnosis based on clinical signs and routine work-ups; (2) the involvement of injuries to other organs, such as spleen, liver, kidney, common bile duct or duodenum; and (3) uncontrolled hemorrhage, sepsis, or even organ failure caused by traumatic pancreatitis and autodigestion of neighboring vascular or visceral structures.

If a pancreatic injury is suspected, a CT scan should be performed. The clinical signs of pancreatic trauma are blurred, and laboratory tests are nonspecific, especially in patients with stable hemodynamics and isolated pancreatic injury. Delayed diagnosis (>24 h after trauma) is associated with higher morbidity and mortality after surgery (17, 18). High-quality CT scans have both sensitivity and specificity of up to 80% in the detection of pancreatic injuries (19). To further assess the severity of the injuries, there are several pancreatic trauma grading schemes, and the American Association for the Surgery of Trauma (AAST) scale is the most commonly used grading system in the literature and higher-grade injuries correlate with poor prognosis (20, 21). According to the AAST scale, the degree of injury is mainly determined by the presence or absence of damage to the main pancreatic duct and the anatomic location of the damage within the gland. For patients with AAST grade III or IV injuries, operative interventions are recommended to decrease morbidity (3). In this case, the initial examination in another hospital suggested pancreatic trauma, and by abdominal CT in our hospital, the injury was further confirmed as AAST grade III trauma with distal (to left of the superior mesenteric vein) transection of the pancreas parenchyma and duct. Therefore, we performed an emergency operation on this patient.

Although the development of minimally invasive techniques has led to the increasing practice of diagnostic laparoscopy, compared with laparotomy, therapeutic laparoscopy has a limited view and working space of the operative field, resulting in the increased possibility of missing organ injury during the operation. Additionally, laparoscopic surgery requires more expertise. Therefore, the adoption of therapeutic laparoscopy in patients with blunt abdominal trauma remains controversial (22–24). However, with the continuous development of imaging technology, accurate preoperative diagnosis of abdominal organ injury can be achieved. In patients with stable hemodynamics, laparoscopic surgery could result in faster postoperative recovery, reduced analgesics, and shorter hospital stays (25–28). In our institution, a tertiary hepatopancreatobiliary center, we have developed significant experience in minimally invasive pancreatic surgery, with an annual volume exceeding 300 pancreatic surgeries, over half of which are performed laparoscopically. This case is our first application of laparoscopic CP for traumatic pancreatic injury. After discussion by a multidisciplinary team that included radiologists, emergency physicians, and pancreatic surgeons, laparoscopy was considered to be safe and feasible.

In the past, DP and splenectomy were standard treatments for patients with traumatic transection of the pancreas neck (29). Compared to DP, CP is a more complex and time-consuming surgical procedure with intestinal reconstruction and pancreaticointestinal anastomosis. Thus, CP patients had higher postoperative complication rates and a longer length of hospital stay (30). However, regarding the long-term outcomes, the overall rates of endocrine insufficiency and new-onset and worsening diabetes were significantly lower in the CP group than in the DP group. Significantly lower rates of exocrine insufficiency were also observed in the CP patients (31). In this case, it took approximately 6.4 h to complete the laparoscopic central pancreas resection, but the intraoperative bleeding was only 100 ml. A biochemical pancreatic fistula (Grade A) was observed after surgery, and the patient was discharged on POD 13. During the follow-up, the patient did not report any clinical manifestation of insufficient endocrine and exocrine function of the pancreas.

Intraoperative angiography with ICG using an NIR system is a useful real-time imaging technology developed in recent years that can improve the performance of surgeons and thus the safety of patients (6). Following intravenous injection, ICG first binds to plasma albumin and becomes fluorescent once excited by NIR light. The fluorescence signal can be visualized by specifically designated cameras and displayed on a screen. These features allow it to reach and highlight only vascularized areas to assist in the real-time estimation of visceral perfusion (32). In previous studies, ICG has been used to verify blood flow, or absence thereof, at the resection margins during colorectal resection, thus allowing the surgeon to change the point of bowel transection at a better-perfused area to prevent anastomotic leak (33, 34). As a relatively hypovascular organ, the perfusion of the pancreas is difficult to assess under white light. As the most common complication after CP, pancreatic fistula is directly related to anastomotic healing, for which adequate blood flow is essential (35). To avoid complications caused by necrotic pancreatic residues and to preserve as much of the normal pancreas as possible, we used ICG angiography to identify the devitalized parts of the pancreas and correct the dissection plane of central pancreatectomy. However, early inflammatory responses, particularly hyperemia due to traumatic pancreatitis, may yield hyperfluorescence signals that do not necessarily indicate viable, healthy tissue. To address this, our approach involved a careful assessment of tissue under both white light and fluorescence modes, combining visual cues to enhance the reliability of viability assessment. Recognizing the elevated risk of pancreatic fistula associated with PJ anastomosis under inflammatory conditions, we employed external pancreatic duct drainage. This strategy has shown potential in reducing leakage risk by diverting pancreatic fluid from the anastomotic site (36–38). To the best of our knowledge, there have been no reports of ICG being used in emergency central pancreatectomy.

This report highlights a novel application of ICG fluorescence and laparoscopic central pancreatectomy (CP) in the context of traumatic pancreatic injury, yet it remains limited by its single-case nature. While the positive outcome observed here suggests that this approach could be valuable, we acknowledge that further validation is necessary. Additional case studies and comparative analyses are required to evaluate the generalizability of these techniques across varied trauma scenarios and to better assess long-term outcomes and potential complications. Future research will be instrumental in establishing standardized guidelines and determining the efficacy of this approach in broader clinical settings.

## 4 Conclusion

In patients with isolated pancreatic injury, emergency laparoscopic central pancreatectomy is feasible when the patient's hemodynamics are stable. This procedure should be supported by a multidisciplinary team and performed by an experienced pancreatic surgeon with advanced laparoscopic skills. ICG imaging technology can help surgeons identify pancreatic tissue lacking blood supply in real time and thus adjust the extent of resection. For blunt pancreatic trauma, the advantages of laparoscopic surgery need to be further confirmed by studies with larger samples.

## Data Availability

The original contributions presented in the study are included in the article/Supplementary Material, further inquiries can be directed to the corresponding author.
